# A qualitative study to investigate Swiss hospital personnel’s perceived importance of and experiences with patient’s mental–somatic multimorbidities

**DOI:** 10.1186/s12888-021-03353-5

**Published:** 2021-07-12

**Authors:** Nicola Julia Aebi, Seraina Caviezel, Rainer Schaefert, Gunther Meinlschmidt, Matthias Schwenkglenks, Günther Fink, Lara Riedo, Thomas Leyhe, Kaspar Wyss, SomPsyNet Consortium

**Affiliations:** 1 Swiss Tropical and Public Health Institute, Socinstrasse 57, 4002 Basel, Switzerland; 2 University of Basel, Basel, Switzerland; 3 Department of Psychosomatic Medicine, University Hospital and University of Basel, Basel, Switzerland; 4 Division of Clinical Psychology and Cognitive Behavioural Therapy, International Psychoanalytic University, Berlin, Germany; 5 Division of Clinical Psychology and Epidemiology, Department of Psychology, University of Basel, Basel, Switzerland; 6 Institute of Pharmaceutical Medicine (ECPM), University of Basel, Basel, Switzerland; 7 Department of Health Canton Basel-Stadt, Division of Prevention, Basel, Switzerland; 8 University of Basel, Geriatric Psychiatry, University Department of Geriatric Medicine FELIX PLATTER, Basel, Switzerland; 9 University of Basel, Center of Old Age Psychiatry, Psychiatric University Hospital, Basel, Switzerland

**Keywords:** Mental health, Mental–somatic multimorbidity, Health care professional, Interprofessional collaboration, Hospital, Qualitative interview

## Abstract

**Background:**

Mental–somatic multimorbidity in general hospital settings is associated with long hospital stays, frequent rehospitalization, and a deterioration of disease course, thus, highlighting the need for treating hospital patients more holistically. However, there are several challenges to overcome to address mental health conditions in these settings. This study investigated hospital personnel’s perceived importance of and experiences with mental–somatic multimorbidities of patients in hospital settings in Basel, Switzerland, with special consideration of the differences between physicians and nurses.

**Methods:**

Eighteen semi-structured interviews were conducted with nurses (*n* = 10) and physicians (*n* = 8) in different hospitals located in Basel, Switzerland. An inductive approach of the framework analysis was used to develop the themes.

**Results:**

Four themes emerged from the data analysis: 1) the relevance of mental–somatic multimorbidity within general hospitals, 2) health professionals managing their emotions towards mental health, 3) knowledge and competencies in treating patients with mental–somatic multimorbidity, and 4) interprofessional collaboration for handling mental–somatic multimorbidity in hospital settings.The mental–somatic multimorbidities in general hospital patients was found to be relevant among all hospital professionals, although the priority of mental health was higher for nurses than for physicians. This might have resulted from different working environments or in efficient interprofessional collaboration in general hospitals. Physicians and nurses both highlighted the difficulties of dealing with stigma, a lack of knowledge of mental disorders, the emphasis place on treating somatic disorders, and competing priorities and work availability, which all hindered the adequate handling of mental–somatic multimorbidity in general hospitals.

**Conclusion:**

To support health professionals to integrate mental health into their work, proper environments within general hospitals are needed, such as private rooms in which to communicate with patients. In addition, changes in curriculums and continuing training are needed to improve the understanding of mental–somatic multimorbidities and reduce negative stereotypes. Similarly, interprofessional collaboration between health professionals needs to be strengthened to adequately identify and treat mentally multimorbid patients. A stronger focus should be placed on physicians to improve their competencies in considering patient mental health in their daily somatic treatment care.

**Supplementary Information:**

The online version contains supplementary material available at 10.1186/s12888-021-03353-5.

## Background

Mental disorders made up 5% of the global burden of disease in 2019 (1), with recent evidence suggesting an even higher burden due to the underestimation of current approaches (2). The World Health Organization’s Sustainable Development Goals (SDGs), particularly SDG target 3.4, underline the importance of treating mental disorders (3). Most general hospitals primarily focus on treating somatic health conditions. However, depressive disorders, anxiety, and other mental disorders are prevalent (4–6). Often, mental disorders are observed as multimorbidity with somatic conditions. For instance, depression is frequently found in combination with coronary heart disease, with the two conditions likely re-enforcing each other (7).

The literature on the prevalence of mental disorders in general hospital settings is limited and not current (8). The prevalence of depression and anxiety in patients in European general hospitals range from 6 to 61% and 11–25%, respectively (4, 9–14). A recent analysis of mental comorbidities in Swiss acute hospitals indicated that 11% of acute hospital patients had received a psychiatric diagnosis (6). These patients typically experienced a longer hospital admission and were more frequently rehospitalized, with the associated increased health care costs (5, 6). Mental multimorbidity was associated with negative progression of the somatic conditions (5), and typically remained undetected or, if diagnosed, neglected (5, 15, 16), which is linked to increased mortality (6, 7). Hence, the early detection and treatment of mental health disorders are crucial (17, 18).

To identify and treat mental–somatic multimorbidity in general hospital settings, a holistic approach is desired (7). However, reviews of this issue have found that patients with simultaneous somatic and mental health conditions receive inadequate care due to health care professionals having low mental health literacy (19, 20) and low confidence in intervening in difficult clinical situations (19). The rather low emphasis on mental health in health professional curriculums is a major factor for this negative outcome. Physicians undergo training with a strong focus on biomedical and technical aspects related to somatic health conditions, especially compared to nurses, who are expected to have interpersonal skills and are trained accordingly (21).

Health care tasks are allocated across the various health professionals based on their educational background, and this shapes the interprofessional collaboration between physicians and nurses. Interprofessional collaboration has been found to positively influence patient outcomes, such as blood pressure and patient satisfaction (22). Likewise, interprofessional collaboration among hospital departments is known to be beneficial (23), and efforts to promote collaboration are encouraged in hospitals. One example is psychosomatic/psychiatric consultation and liaison (CL) services, which mediate between somatic wards and mental health specialists, providing physicians in general hospitals with advice on the patient’s psychosocial issues and how to handle them (24). However, insufficient referrals to the CL service have been observed (25, 26), highlighting potential barriers to integrating mental health care into general hospital settings.

It is, therefore, necessary to better understand the hospital personnel’s view of mental–somatic multimorbidity in general hospitals. Hence, this study investigated hospital personnel’s perceived importance of and experiences with patients having mental–somatic multimorbidities in general hospital settings in Basel, Switzerland. In addition, we explored differences between physicians and nurses regarding the management of mental–somatic multimorbidities in general hospitals.

## Materials and methods

### Study setting

This qualitative study was conducted in Basel, Switzerland, in three general hospitals (University Hospital Basel, Bethesda Hospital and the University Department of Geriatric Medicine FELIX PLATTER). These institutions are part of a project called SomPsyNet (27), which aims to prevent the consequences of psychosocial distress of patients in somatic acute hospitals by establishing a collaborative care network. To this end, these hospitals are implementing a stepped and collaborative care model in the daily hospital routines of selected wards to more appropriately and effectively identify and address the psychosocial burden of patients admitted for somatic conditions.

The University Hospital Basel and the University Department of Geriatric Medicine FELIX PLATTER are involved in teaching and research. The latter focuses on acute geriatric medicine, geriatric psychiatry, and rehabilitation. The Bethesda Hospital is a private hospital focusing on gynecology and rehabilitation. Regarding patient volumes, 5365 patients were discharged from the University Department of Geriatric Medicine FELIX PLATTER (28), 38,570 from the University Hospital Basel (29), and 6062 from Bethesda Hospital (30) in 2019.

This study was approved by the Ethics Committee of Northwest and Central Switzerland (EKNZ; ID Req-2019-01219). All the interviews were conducted upon written informed consent.

### Study population

Three categories of health professionals were interviewed: nurses, physicians, and hospital administration personnel such as project and data managers, and IT specialists. Three interviewees, one psychologist and two psychosomatics, belonged to the CL service team to obtain their input on the collaboration with other hospital departments. Due to our interest in the nurses’ and physicians’ perspectives, data from health administration personnel were excluded from this analysis. To guarantee privacy, we included the data of the psychologist with the nurse group. The health professionals represented different hospital services: gynecology, rehabilitation, rheumatology, internal medicine, and psychosomatics. All interviewees were involved in SomPsyNet, either in the planning or later in the implementation. The first author (NJA) contacted the SomPsyNet project team and the hospital ward line managers to request the contact information of potential interviewees differing in age, gender, and job position. In this way, various perspectives within the professional groups were included. The potential interviewees were then contacted by email. The interviewees and NJA did not know each other before the interviews.

### Data collection

The interviews were conducted using a semi-structured interview guide (Supplementary 1) developed based on the literature and discussions within the research team. After pilot-testing with three former nurses, the interview guide was adapted to focus on four main topics: 1) knowledge about mental health in somatic patients, 2) experiences with the mental health of somatic patients, 3) clinical processes at the hospitals regarding patients with mental–somatic multimorbidity, and 4) personal attitudes towards the mental health conditions of patients treated for somatic health conditions. The semi-structured interviews were conducted between February and July 2020 prior to the launch of the new mental health-focused project, SomPsyNet. Due to the SARS-CoV-2 pandemic, six interviews with physicians were conducted over the phone. All other interviews were conducted in person at the interviewee’s workplace, in a location where they felt comfortable. To be able to speak openly, the interviews were conducted in Swiss German or German, depending on the interviewee’s preference. All interviews were audio-recorded and conducted until the information provided was redundant. Developing the interview guide, conducting the interviews, and analyzing the data were carried out by NJA, a female epidemiologist who has attended several qualitative research courses.

### Data analysis

The interviews were transcribed verbatim and coded in NVivo 12 (31). Based on an in-depth reading of the transcripts, codes and themes for inductive analysis were developed. Framework analysis (32) was used to extract the perceived importance of and experiences with mental health in somatic patients, because this analysis method enabled a comparison of the professional groups. The seven steps recommended by Gale et al. were followed: 1) transcription, 2) familiarization with the interview, 3) coding, 4) developing a framework, 5) applying the framework, 6) charting data into a framework matrix, and 7) interpreting the data (32). To guarantee reflexivity, NJA kept detailed research notes and had discussions with her supervisor (KW). The research notes included reflections after each interview, which were consulted during the analysis. Due to the high workload of health professionals and resulting limited availability, we did not conduct member checking. Reporting was guided by the Consolidated Criteria for Reporting Qualitative Research (COREQ-32) (33).

## Results

In total, 18 semi-structured interviews were conducted, with an average duration of 30 min each (15–46 min). The professional groups displayed similar demographic characteristics, except that most of the interviewees were women and the nurses had more professional experience than the physicians (Table 1).

**Table 1:** Demographic characteristics, affiliated institutions and duration of interviews (*n* = 23)

Characteristics	Physician (*n* = 8)	Nurse (*n* = 10)*
Age [years]		
Mean (SD)	38.8 (10.2)	43.4 (13.5)
Range	28–59	26–62
Sex [N]		
Female	5	8
Male	3	2
Years in profession		
Mean (SD)	10.1 (10.1)	18.5 (13.4)
Range	3–32	4–35
Hospital [N]		
University Hospital Basel	4	7
Bethesda Hospital	4	3
Department [N]		
Rheumatology	1	1
Rehabilitation	1	1
Internal Medicine	3	6
Gynecology	1	1
Psychosomatics	2	1
Duration of interview [min]		
Mean (SD)	26.6 (7.5)	34.2 (9.6)
Range	14.6–35.4	19.0–46.3

The table represents the number of interviewees in categorical variables (sex, hospital, department) and the mean, standard deviation (SD) and range of continuous variables (age, years in profession, duration of interview).

*including one psychologist belonging to the CL service

Among the 18 interviews, four themes regarding general hospital settings were extracted inductively: 1) relevance of mental–somatic multimorbidity within general hospitals, 2) health professionals managing their emotions towards mental health, 3) knowledge and competencies in treating mental–somatic multimorbidities, and 4) interprofessional collaboration for managing mental–somatic multimorbidities within general hospitals.

### Relevance of mental–somatic multimorbidities within general hospitals

Mental–somatic multimorbidity was defined in the interviews as somatic patients with any kind of mental health issue. The prevalence of mental–somatic multimorbidity among somatic patients admitted for hospital care was perceived to be high. Medical events, such as requiring a visit to an emergency department or receiving a cancer diagnosis, were described as having a large impact on patients’ mental health. Only one physician stated that encountering mental–somatic multimorbidity in their daily routine was infrequent, however, they admitted that these conditions might remain unrecognized.

*“Well, speaking in relative terms, we have lots of patients, but I think that relatively few patients actually have a mental disorder or stress. Obviously, maybe we don’t recognize them.”* physician, age 32, male

However, all interviewees agreed on the importance of mental health in general, although its priority may depend on the professional group and the specialty they work in. While mental health was a high priority for nurses, it was lower for physicians.

*“I do believe that for nurses it (mental health) is of greater importance, since it is them who have a lot of contact with the patients and must deal with the various emotions”* physician, age 42, female

The separation of body and mind was perceived by many as artificial because they had observed the impact that mental health conditions could have on somatic symptoms, signs, and treatment.

“*Let’s take for example oncology patients that are often confronted with pain. If one focuses on the somatic side of things, the patient will receive very high dosages of pain medication. This also occurs with conventional medicine physicians. With more experience one might be able to notice, or others around you make you aware of it, that there is a strong psychosomatic side to it and when one tries to remove a patient’s fears and worries, this actually contributes to decreasing the overall painkiller prescriptions, although not much has changed at the somatic level.”* physician, age 43, male

Concurrently, these quotes emphasize the effect that mental health conditions could have on a patient’s hospital stay. As stated by our interviewees, patients suffering from mental–somatic multimorbidity were less likely to adhere to their somatic conditions treatment, leading to lower treatment success. The nursing staff further described patients as *“difficult”* because *“the patient does not do what we (the nursing staff) want them to do”* (nurse, age 43, female). Thus, they emphasized that more effort and time, a scarce resource in this setting, were needed to treat patients with mental–somatic multimorbidity. This situation also applies to the time before the mental health condition is diagnosed. Physicians have experienced the challenge of finding an explanation for some patient’s somatic complaints.

*“Exactly, but I do believe that this notion is often in the back of the mind of the assistant physician. Only when they have done everything they can possibly do and have considered various options and there is nothing that can possibly match, then one is glad to be able to see further if it might be psychological or due to pressure or something similar.”* physician, age 42, female

The recognition of mental–somatic multimorbidity could depend on the length of hospitalization. According to our interviewees, the duration of hospitalization affects the recognition of mental–somatic multimorbidity in different ways. First, the longer the patient stays, the more likely that symptoms of mental health conditions will evolve. Therefore, patients hospitalized for orthopedic procedures with a comparatively short hospital stay are less likely to display mental health symptoms than patients admitted for a longer term in internal medicine. Second, physicians and nurses have a greater chance to recognize mental symptoms in cases of longer treatments and hospital stays. Even if patients try to hide their feelings, with a longer duration of care, they might build up trust and report certain concerns. However, most interviewees reported not being able to efficiently use the duration of hospital stay.

*“It can also be extremely exhausting since our daily work in our unit barely allows us to have the necessary time to adequately treat and help patients.”* nurse, age 37, female

In particular, physicians’ high workload hindered the adequate recognition and treatment of patients with mental–somatic multimorbidity in general hospitals. Time constraints led to lowering the priority of mental health conditions in these settings.

*“With such a tight work schedule, it’s easier to prescribe a blood sample analysis or, as an example in the case of an oncology patient, to increase their painkillers, rather than conducting a longer conversation, where anxieties may be discussed.”* physician, age 43, male

In addition to the limited time, insufficient privacy and tranquility was highlighted by the nurses.

*“[ … ] we are in the room, talking (with the patient). There is always somebody entering the room ‘Could you … ’ or we have hospital rounds or must answer the phone. Especially with such diseases, I think that tranquility and being able to sit at the bedside and just talk (to the patient) without being constantly interrupted are the most important.”* nurse, age 29, female

Another reason for the late recognition of mental health conditions by health professionals could be social norms. Most interviewees described mental health as a taboo in society, although this deviated from their personal view. Depending on age, gender, and culture, patients have not been talking about their mental health because this is often seen as a weakness or failure. The societal view could affect the physicians and nursing staff by increasing their anxiety and inhibit them from actively bringing up mental health issues.

*“Suicide risk is a topic that is inherently connected to a lot of fear, and that when one dares to talk to other people (patients) about it … there is a deep inner fear.”* nurse, age 55, female

*“Most of the time, the questions are related to fears. We always are afraid to talk about such things, about ‘Oh, now, I cannot talk to a patient about psychosomatics or psychiatric issues or sexual issues or death or similar taboos.’ I sometimes realize that these are our own fears. If we bring these up with patients, their willingness to talk about it is high.”* physician, age 43, male

### Managing emotions towards mental health conditions in general hospital settings

Various emotions of the health professionals related to the patients’ mental health and working with these patients were described, such as difficulties in understanding the patient, difficulties in maintaining a professional attitude, powerlessness, uncertainty, anger and the feeling of being left without support. These emotions arose in situations with patients but also in interactions with other health professionals.

The medical staff of the included hospitals has several possibilities to talk about their concerns and experiences with handling mental health problems among patients. First, the line manager can offer support to the nursing staff. If a nurse is suffering mental health issues such as anxiety, the line manager will try to alleviate their duties and find them appropriate support. Similarly, if difficulties with patients occur, the line manager is supportive.

*“In everyday life, one realizes that it can be momentarily quite difficult and when patients simply do not do what we would like them to. This can cause one to be angry and storm out—there are such moments. We have line managers in our department that are responsible; when this occurs, they say ‘Yes, that is just another additional problem that we have to look at’.”* nurse, age 43, female

Second, talking to mental health specialists helped the interviewed health professionals to deal with the emotions, especially when working with *“difficult patients”*. Sometimes, the mental health specialist joined the team meetings to explain the patient’s manipulative or aggressive behavior, leading to a better understanding by the nursing staff.

*“When the patient receives a diagnosis or it is otherwise understood that he has always been this way, I don’t have the pressure that this behavior has to stop now. It is then mostly trying to tolerate the situation somehow.”* nurse, age 59, male

Third, the exchange with team members or private contacts who also work in health care is important for health professionals to manage their own emotions. These exchanges can offer some reflections on patient situations.

*“I am also somebody who needs extra reassurance. Was it ok or not how I handled it? I will also ask for advice because I have an unsure feeling and I am aware that it can always have been dealt with better or simply differently. I would like support such as ‘Yes, it was fine the way you did it’ or ‘This and that could be done differently next time’.”* nurse, age 26, female

This support can occur through interacting with colleagues who have the knowledge and competencies needed to treat patients with mental health conditions.

### Knowledge and competencies in treating mental–somatic multimorbidity in patients admitted for a somatic conditions

Knowledge about mental symptoms, and competencies in handling multimorbid patients were mentioned to be influenced by several factors that affect the detection of mental health conditions in general hospitals. Physicians and nursing staff both have had a strong focus on somatic issues because this is typically the primary reason for hospitalization. Therefore, physicians only considered mental components *“if lots of somatic issues are excluded”* (physician, age 42, female). This blind spot was already forming during their education and training.

*“[...] due to our background, we aren’t competent to always include both (somatic and mental health conditions)”* physician, age 43, male

As emphasized by these quotes, the training of nurses and physicians concentrates more on somatic conditions than on mental health. Reasons for this insufficient education and training were explained through *“little evidence-based methodology”* (physician, age 33, male) on mental health conditions in general hospital settings and through a lack of sensitivity towards mental health. Although one physician observed a change in sensitivity, it was highlighted that time is needed to integrate mental health into the curriculum. During training, the nursing staff has to decide early on what their educational focus will be (psychiatry, acute somatic, or long-term care), limiting the access to knowledge and competencies related to mental health in somatic care. As nursing staff mentioned, despite partially learning how to handle these patients, it differed from reality.

*“It has been discussed practically and theoretically how you should proceed in such a case. Nonetheless, I do believe that when confronted with reality it is very different.”* nurse, age 26, female

Mock situations during education and training were not able to mirror the behavior of patients, which influenced the identification and acceptance of mental–somatic multimorbidity. Interviewees mentioned perceiving various patient behaviors: While some patients were perceived as manipulating nursing staff and playing nurses off against each other, other patients were perceived as masking their feelings. Therefore, sensitivity for trivial statements and symptoms is essential, although it can be overwhelming in the beginning.

*“I have always tried, which is something I also tell the nurses, to be extra attentive like an extra-terrestrial with many antennae picking up signals. Even if one is doing something small, such as measuring blood pressure, changing the infusion bottle or something quite routine, you should always enter the room with these antennae trying to sense what else is going on.”* nurse, age 56, female

Despite limited time with the patients, most interviewees emphasized the importance of communication and informing patients about their health status and the further actions to be taken. Reassuring conversations could help patients to calm down in escalating situations. Moreover, it is important to listen to the patients and *“believe that this, what this person says, in fact, has now any justification or truth”* (physician, age 43, female). Physicians stated the importance of taking patients seriously and communicating clearly.

*“One often reduces, a bit, existing prejudices; in particular, psychiatric problems. People say ‘Yes, well I am not crazy or anything’. There is a false concept of what psychiatric or psychosomatic questions are, and what lies behind them. One tries to bring more awareness and clarity to this false concept.”* physician, age 43, male

Due to these misconceptions, patients were sometimes not willing to open up. Hence, one nurse suggested offering the patients to talk to them at another time or to speak with other nursing staff, demonstrating the importance of collaboration within the hospital.

### Interprofessional collaboration for managing mental health conditions in general hospitals

Nurses seem to be “*more and more on eye-level”* (nurse, age 62, male) with physicians. Still, nurses described the interprofessional collaboration between nurses and physicians as mixed. While some physicians value nurses’ opinions, others still see them as “auxiliary” staff. This led to nurses repeatedly pointing out potential mental–somatic multimorbidity while feeling left on their own.

*“[...] because most often they (nursing staff) recognize these things (mental health conditions) and then, they seek out help with no response. They feel that nobody cares. There’s a problem here.”* nurse, age 56, female

However, especially with complex patients, the physicians stated that they relied on information from the nurses because nurses spend more time with the patient. The interprofessional collaboration between nurses and physicians, therefore, depends on both professionals.

*“We have doctors with whom collaboration is excellent. They recognize it (mental health conditions) well. [...] But if they run into an ignorant nurse, then it progresses just as little as vice versa.”* nurse, age 62, male

Nonetheless, the physicians decide upon the course of action taken and whether, for instance, the psychosomatic/psychiatric CL service—the main route of interprofessional collaboration between the different wards and the mental health professionals—should be involved. Either the physicians recognize the necessity of consulting with the mental health specialist or the nursing staff notify them of this need, because nurses recognize it due to closer patient contact. After the consultation, the physicians receive feedback, including treatment recommendations if necessary.

Most physicians rated this way of collaboration as efficient. Nevertheless, others questioned the fact that specialties have been separated and the lack of knowledge about mental–somatic multimorbidity among physicians, because the physicians might overlook important information. In addition, they might not be able to ask precise questions, diminishing the psychosomatic / psychiatric CL service’s efficiency.

*“[ … ] this is maybe our fault or flaw. We are poorly trained for these kinds of questions. We cannot ask good enough consultation questions that allow us to get the answers (by the CL service) that we want.”* physician, age 43, male

The nursing staff highlighted other critical aspects about the psychosomatic/psychiatric CL service—the nursing staff cannot trigger a consultation on their own, the wait until patients receive medication or other support can be too long, and sometimes too many people are involved in the process.

*“It can take around 2-3 days until it’s filled out and around a week or more until the psychologist can arrive. If they also need medication, it can take up to 2-3 weeks until they start to feel the effects. So, all in all, it is an extremely long process until things start to look up.”* nurse, age 43, female

*“There are drawbacks if too many people start to get involved, such as a decreased quality of the inter- and intra-disciplinary communication. If too many people are pulling on various threads and have a say, it becomes too much.”* nurse, age 37, female

One nurse mentioned that mixing the somatic and mental health staff on the wards could lower the obstacles nurses face in convincing physician about the need for a consultation with the mental health specialist. As one interviewee mentioned, something similar has already been in place in one of the hospitals, for example, the liaison service where some psychologists are employed at a specific ward. In cases where support is needed by the psychosomatic/psychiatric CL service, this psychologist can take over.

At one hospital, rehabilitation and rheumatic wards have weekly interdisciplinary team meetings in which attending physicians, psychiatrists, physiotherapists, case managers, and the nursing staff are present to discuss each patient. However, due to time constraints, instituting interdisciplinary team meetings in all wards has not been possible.

*“It would be ideal to have an interdisciplinary relationship between departments. Unfortunately, this is not necessarily possible in every department. First, counselling doesn’t occur all that often and it takes up a lot of time passing when we cannot treat other patients. One has to take into consideration the economic means. It would naturally be ideal to have a particular time set aside to have the opportunity to talk, but this, of course, does not always work in a daily-life.”* physician, age 59, female

Similarly, case conferences have been conducted in some wards and hospitals. Here, physicians and/or nurses present a specific patient who concerns them. By contrast, in interdisciplinary team meetings, all patients are discussed. The case conferences took place within the same professional group and wards, and also between different professional groups and wards, increasing the sensitivity of the hospital staff.

*“During case conferences, often ethics problems are left out and one rather looks into the nursing process. We will assess whether anything was missed or whether the supposition that we have about the particular patient is, and on this basis sensitize the health professional ‘Aha, there is more than, mobilizing, washing, nursing, hair-drying and such things.’”* nurse, age 62, male

The nursing staff and three physicians further described different types of informal collaborations, such as exchanges within the team and with people in the private setting who have the same job. A functional team was characterized as a space where problems, anxieties, and worries are shared with others, leading to exchanges about treatment strategies or support for each other, for instance, by taking over a patient.

*“It is my belief that the manifestation of a functioning team is when one can freely express own necessities and this is positively perceived by one’s colleagues, and in turn, one offers help.”* nurse, age 31, female

Exchanges within the team are particularly relevant if some team members lacked understanding of the *“difficult patient*” and thus exhibited unprofessional behavior.

*“The following is expressed in dissatisfaction, where they don’t want to take care of the patient anymore since they don’t get along. Negative things are said, which one hardly wants to repeat, which is truly unfortunate. There are many negative reactions that can manifest themselves.”* nurse, age 37, female

However, such exchanges did not take place in all teams. In particular, physicians felt that the pressure of establishing their careers and their lack of sensitivity towards mental health limited their exchanges.

*“Well I think that is not much of a topic; simply, as we previously described, due to an outdated image. Also in psychology, as at the Center Hospitals, one is exposed to a certain pressure, especially the young doctors, who still have to establish themselves. Particularly there, it is a little bit difficult to discuss such things. Be it from one's own personal experience or be it also that one wants to out oneself to have a particular sensitivity for such questions. So the tone is usually more offhand.”* physician, age 43, male

## Discussion

Mental–somatic multimorbidities were generally rated important and relevant in general hospitals, although nurses gave more weight to the mental health dimension than the physicians did. Effective and efficient handling of mental health conditions among somatic patients faces various challenges, including the strong focus of hospitals on somatic conditions, the absence of sufficient knowledge and competencies for dealing with mental health problems, and weak interprofessional collaboration.

### Relevance of mental–somatic multimorbidity within general hospitals

The importance of mental health in general hospital settings is highlighted by the perceived high frequency of mental–somatic multimorbidity among patients. This is in accordance with cross-sectional studies (4, 8–14), although the literature on the prevalence of depression in general hospitals is fragmented, and previous studies are not conclusive (8). However, it must be assumed that health professionals cannot identify the full range of multimorbidities for a variety of reasons.

In Switzerland, Rentsch et al. (11) stated that only half of depressive patients are detected, which is in line with the low recognition of mental health conditions in hospital settings in other studies (34–37). Obstacles to recognizing mental disorders are the patient’s age, personality traits, and the severity of the mental issues (34). Further, the recognition is dependent on the age and specialty of the physician (38). In addition to barriers such as stigma and a lack of knowledge and sensitivity, the physicians’ high workload is a strong barrier to recognizing mental–somatic multimorbidity. Physicians particularly encountered strong limits to their availability impeding their contact time with patients and interprofessional collaboration with other health professionals. Hence, combined with the pressure to establish a somatic-based career, the time constraints lead to inadequate recognition of mental–somatic multimorbidity.

An additional challenge is the strong focus on treating somatic health conditions at hospitals to the detriment of treating mental health conditions in this setting. This strong focus on somatic health conditions leading to insufficient access to mental health services in general hospitals was even stated by patients diagnosed with a personality disorder (39). Previous studies described that health professionals working in a general hospital do not see mental health conditions as belonging to their competencies and tasks (19, 40). On the one hand, this could be triggered by the high workload, reducing the time available for such tasks. On the other hand, as mentioned by some interviewees in our study, this strong focus begins during health professionals’ early education, leading to a lack of knowledge and competencies.

The lack of knowledge and competencies was described as a major barrier to mental health treatment in a somatic setting (19) and was mentioned by some physicians in our study. However, this lack may depend on the specialty: where major life changes, such as a cancer diagnosis, are seen to have large impacts on mental well-being, a more holistic approach is desirable. Furthermore, a lack of knowledge could lead to more negative attitudes (41), highlighting the importance of education and training. Nonetheless, younger nurses mentioned that theory and practical situations differ greatly, impeding optimal preparation to work with patients suffering from mental–somatic multimorbidity. These difficulties may arise due to the unpredictable behavior of the patient.

### Nurses’ and physicians’ differing perspectives on mental health in general hospitals

Patients’ unpredictable behavior is related to the lack of adherence to suggested treatments and to the difficulties in handling these patients because these patients may not follow the nurses’ directions. In this regard, nurses reported dealing with *“difficult patients”*, which has been emphasized by others (19, 42, 43), indicating that negative stereotypes remain. One possible reason why only nurses perceive a patient as *“difficult”* may be the increased time they spend at the bedside compared to physicians. According to Giandinoto et al. (42), this perception is related to the somatic hospital setting not being appropriate for multimorbid patients suffering from mental and somatic health conditions, because the patient’s adherence is diminished, and the hospital environment appears to be insufficient.

In our study, a suboptimal environment was mainly emphasized by nurses. They stated the great importance of offering a calm and private room for discussions of the patient’s mental health. However, this environment is not available in all wards. For instance, busy emergency department does not have the time or space to discuss sensitive matters. Similarly, other somatic settings have difficulties owing to insufficient infrastructure (19), such as noisy places or places that lack privacy (26). While physicians can sometimes take the patient to a private room and talk without interruptions, nurses typically do not have this opportunity despite their considerable interest in supporting patients with a more holistic approach.

The physicians more frequently made referrals for patient support for mental health conditions without explicitly communicating the situation with their colleagues. On the one hand, this could be due to time pressure and the historical view of their superiors and other colleagues, leading to the pressure to concentrate on somatic conditions. On the other hand, the described societal view of mental health may lead to fear of addressing mental health with the patient. Other studies observed that fear regarding patients’ unpredictability impeded adequate treatment (42).

While these factors can lead some physicians to be hesitant to talk about mental health in general hospital settings, other physicians might be reluctant to integrate mental health issues at all. The physicians’ strong focus on the patient’s medical condition (44) and differences in the duration of work experience (19), might lead to see mental health not as part of their business (40) and, in turn, to physicians’ hesitancy in integrating mental health in somatic hospital settings.

### Interprofessional collaboration for managing mental health conditions

As observed in our study, different routes of interprofessional collaboration are possible, such as team meetings across health professionals, either formal or informal, and psychosomatic/psychiatric CL services. The latter has been shown to to improve patient outcomes (22). Nevertheless, the interviewed physicians emphasized that interprofessional collaboration could be inefficient due to a lack of knowledge. According to previous studies, the lack of knowledge and competencies (45) and the not recognizing mental health conditions (38) leads to reduced referrals to psychosomatic/psychiatric CL services, supporting our results that a lack of knowledge is an important barrier to identifying mental health conditions in general hospitals. Other barriers to referrals to psychosomatic/psychiatric CL services were time pressure and poor communication among mental health professionals (38).

Open, transparent, and regular communication between nurses and physicians was seen to facilitate interprofessional collaboration (46). However, in our study, the nurses described some physicians not accepting nurses’ views. Similar observations were reported by another Swiss study (16). Differing perceptions of collaboration might be one reason—while nurses see their competency in supporting decision-making regarding the patient’s treatment, some physicians still perceive them as “auxiliary” staff (47–49). Another reason for the differing perceptions of nurses and physicians is their educational background. Whereas the educational focus of physicians is biomedical knowledge and technical skills, nurses are also trained in interpersonal skills, including working in a team (21). These interpersonal skills might enhance the nurses’ ability to adequately recognize and treat patients with mental–somatic multimorbidity in general hospitals. Overcoming communication barriers would increase trust and respect, thereby enhancing effective collaboration between nurses and physicians. Still, structural barriers such as contact times between health professionals might impede this transformation.

The frequency of interactions and time constraints build different ward cultures that, in turn, influence collaboration (47). Time pressure, unclear role and task descriptions, and poor organization were barriers to interprofessional collaboration between nurses and physicians (46) as well as between somatic and mental health specialists (40). This might amplify challenges in communication, further leading to unrecognized mental health conditions. However, Jasmin et al. (50) observed an improvement of interprofessional collaboration with time.

### Strengths and limitations

This study has several methodological advantages. Nurses and physicians from different hospitals in the same canton were interviewed. One of the hospitals implements a mixed system. This hospital is run by a chief physician who cooperates with affiliated ambulatory attending physicians, giving a broader view into differences in experiences with mental health and interprofessional collaboration. This provided information about the potential scale up of mental health projects in general hospital settings.

However, this study also has some limitations. The recruitment strategy involved line managers proposing the interviewees. The hospital personnel’s experiences with and perceived importance of mental health in general hospital settings might therefore be limited. As the nurses mentioned, some colleagues had less understanding of mental health issues in the somatic setting. One direction of future research should be to study these health professionals to assess the reasons for their feelings and behavior, and evaluate how changes could be made.

Further, the included wards do not represent the full range of hospital wards. For instance, surgery departments with rather short hospital stay durations might place low importance on mental health because they may not be as confronted with these issues as, for example, an internal medicine ward is. Challenges occurring with patients having severe mental disorders, such as schizophrenia or bipolar disorders, were not explicitly mentioned by our interviewees. Also, there was no mention of issues related to suicide ideation or attempts, which may be partially explained by the fact that patients specifically presenting with related conditions are commonly hospitalized in acute psychiatric hospitals. Future research in Swiss general hospitals may consider focusing on these patients.

As with all interview studies, we cannot exclude the possibility that interviewees gave socially desirable answers. However, we stressed the importance of conducting the interviews in a place where the interviewees felt comfortable. This was highlighted for in-person and phone interviews. Some interviews took place in a cafeteria, and people who were not involved in the study were able to enter the room. Nevertheless, the interviewees did express critical views, indicating that they felt comfortable and spoke openly.

Considering that this study was launched right before the SARS-CoV-2 pandemic in Switzerland, we cannot exclude effects of the pandemic on the views expressed by the health professionals. On the one hand, during this time, the importance of mental health was widely discussed, and health professionals’ attitudes towards patients with mental–somatic multimorbidity could have been positively influenced. On the other hand, the health care system switched its focus from non-communicable diseases to communicable diseases. Therefore, the perceived importance of mental–somatic multimorbidity could have been diminished.

## Conclusion

These findings suggest that mental health conditions among hospital patients being treated for a somatic conditions were seen to be frequent. Furthermore, the need to adequately address and deal with mental–somatic multimorbidity was perceived to be high by hospital staff. The interest in integrating mental health issues in general hospitals seemed to be higher for nurses than for physicians. However, some of the nurses’ views of patients show that negative stereotypes of mental conditions still exist. Moreover, structural and communication challenges were apparent, impeding the adequate treatment of mental–somatic multimorbidity in general hospital settings.

Offering an appropriate environment for handling multimorbid patients in a calm and private setting should be promoted. Further, strengthening interprofessional collaboration and improving knowledge and competencies related to mental–somatic multimorbidity are essential to improve patient health outcomes and to address negative stereotypes, and should be prioritized during education and continuing training. Physicians should be particularly targeted by awareness and educational programs to encourage them to integrate mental health treatment in general hospitals.

## Supplementary Information

**Additional file 1.** Interview Guide: mental health in general hospitals

**Additional file 2.** SomPsyNet Consortium

## Data Availability

The dataset presented in this article are not publicly available, because it contains information that could compromise the privacy of the interviewees. Requests to access the dataset can be directed to the corresponding author.

## Abbreviations

CL service: consultation and liaison service
SDG: Sustainable Development Goals
SomPsyNet: comprehensive healthcare project for patients from SOMatic hospitals that promotes the prevention of PSYchosocial distress by establishing a stepped and collaborative care NETwork in Basel, Switzerland
