# Supplementary material for: A qualitative study to investigate Swiss hospital personnel’s perceived importance of and experiences with patient’s mental–somatic multimorbidities
Source: BMC Psychiatry. 2021 Jul 12;21:349. doi: 10.1186/s12888-021-03353-5 (PMC8274261; doi:10.1186/s12888-021-03353-5)
Supplement: Supplementary file 1 — Additional file 1. Interview Guide: mental health in general hospitals [file 12888_2021_3353_MOESM1_ESM.pdf]

Interview Guide: mental health in general hospitals

Information

Hi, thanks for taking the time to talk to me.

- Presentation of interviewer (NJA)
- Informed consent
  - Aim: How is mental health integrated on the hospital wards?
  - Approved by ethics committee
  - Participation is voluntary and can be withdrawn at any time.
  - The interview is recorded and will be transcribed.
  - Data are encrypted and password-protected.
  - Only authorized persons have access on un-encrypted data and these people are bound to secrecy.
- There are no right or wrong answers, but interested in collected experiences and impressions.
- Indicate if you don't want to answer a question.
- Are there any questions?
- Sign consent
- Turn on audio-recorder

Introduction

1. What is your profession?
   - How long have you been working in this profession?
   - What do you like most about this profession?
   - What do you dislike about this profession?

Knowledge about/experiences with mental health of patients

2. Have you ever been confronted with people who were suffering from physical and mental conditions?
   - Could you please tell me more about that?
   - At work? In private?
   - How was this for you?
3. Have you ever had any experiences with somatic patients who also suffered from a mental condition during your work? (only health professionals)
   - Could you please tell me more about such a patient?
   - How was this for you?
   - How did you behave in this situation?
   - Have you had any support by others?
   - Did you change anything in the treatment of this patient?
   - How would you describe these patients?
   - What is the best part when working with these patients?

- What is the worst part when working with these patients?

4. Were you ready to handle somatic patients who have mental conditions?
   - How did you prepare yourself for these patients? / What was missing to be prepared for these patients?
   - What would you have needed to be prepared for these patients?

Processes of somatic patients with mental conditions

5. Is there a standard procedure in case of a somatic patient additionally suffering from a mental condition?*
   - What does this procedure look like?
   - Who do you inform if you think that a patient is suffering from a depression or anxiety?
   - When do you inform [the above mentioned person]?
6. What do you like the most about this procedure?
7. What do you like the least about this procedure?
8. Which role do you have regarding communication between the wards? (only administration personnel)
   - What do you like about this?
   - What do you dislike about this?
9. How does the communication between the wards work if, for instance, problems are arising? (only administration personnel)
   - Who should you approach?
   - What happens next?
   - What do you like about this?
   - What do you dislike about this?
10. Are there any plans for changes in handling somatic patients who have mental conditions such as depression or anxiety? Which ones?
    - From your point of view, what is the purpose of these plans?
    - Have you ever heard about SomPsyNet? What? When? In which context?
    - What is your role in this project?
11. Which role did or do you have in the planning of SomPsyNet? (only personnel who were involved in planning)
    - Could you please tell me more about this?
    - What do you like the most about the planning of this project?
    - What do you like the least about the planning of this project?
    - What would you do differently, if you could plan the project?
    - What would you do the same way, if you could plan the project?
    - What are the differences of SomPsyNet with other projects?

- What are the similarities of SomPsyNet with other projects?

12. Why do you think is the testing of mental conditions such as depression and anxiety implemented?
13. What is the priority/emphasis of the testing of mental conditions such as depression or anxiety in comparison with other tasks you have?
14. Do you think that the testing and treatment of somatic patient with mental conditions such as depression or anxiety could lead to better results? Be it for patients, but also physicians, nurses or the health system?
15. Do you or your colleagues have any problems with testing patients for mental conditions? Which ones?
16. Within this project, somatic patients are asked questions about mental health: How do think the patients will react to these questions?
    - Could you please tell me more about this?
    - What are examples for positive reactions?
    - What are examples for negative reactions?

Personal view

17. What would you say: Which role does mental health play in somatic patients?
    - Are you talking with someone about this topic? Colleagues? Family? Friends?
    - What are you talking about?
    - When do you talk about this?
    - How often do you talk about this?

Closing

18. Is there anything else you would like to add about this topic of mental health or the procedures at the hospital?

[turn off audio-recorder]

Again, I would like to thank for your time. It was an interesting and informative discussion. If you have any questions or anything to add later, you can contact me. You can find my contact dates on your copy of the informed consent form.
