# Supplementary material for: A qualitative study to investigate Swiss hospital personnel’s perceived importance of and experiences with patient’s mental–somatic multimorbidities
Source: BMC Psychiatry. 2021 Jul 12;21:349. doi: 10.1186/s12888-021-03353-5 (PMC8274261; doi:10.1186/s12888-021-03353-5)
Supplement: Supplementary file 2 — Additional file 2. SomPsyNet Consortium [file 12888_2021_3353_MOESM2_ESM.pdf]

SomPsyNet Consortium

Nicola Julia Aebi[1,2], Seraina Caviezel[3], Rainer Schaefert[3], Gunther Meinlschmidt[3,4,5], Matthias Schwenkglenks[6], Günther Fink[1,2], Lara Riedo[7], Thomas Leyhe[8,9], Kaspar Wyss[1,2], Klaus Bally[10], Alexander Frick[3], Iris Bänteli[3], Anja Studer[7], Marco Bachmann[11], Sibil Tschudin[12], Andreas Dörner[13], Christina Karpf[7], Gabriele Bales[14], Katharina Sophie Barthelmess[3], Stefano Bassetti[15,16], Reto Baumgartner[17], Stefanie Bosman[13], Virginie Bourquin[3], David Büchel[3], Luka Damjanov[3], Lukas Ebner[3], Jennifer Erb[3], Peter Ettlin[18], Elvira Fasel[11], Lavinia Flückiger[19], Johanna Fremmer[3], Florian F. Grossmann[20], Anja Hermann[21], Matthew Hotopf[22], Lydia Isler-Christ[23,24], Maria C. Katapodi[25,26], Robert C. Keller[27], Sabrina Klimmeck[28], Melinda Kress[3], Yvonne Künstle[3], Undine E. Lang[29], Yasmin Liechti[3], Sherado Mazander[30], Daria Meier[3], Alexander Minzer[31], Francisca Schiess[32], Felix Schirmer[33], Nadine Schur[6], Peter Schwob[34], Sonja Seelmann[15], Gayoung Son[3], Thomas Steffen[35], Friedrich Stiefel[36], Marion Tegethoff[37], Corinne Urech[38], Thomas von Allmen[39], Lilly-Sophie Walzer[3], Sybille Werner[3], Andrea Wetz[40], Dragana Weyermann[41], Christoph Zäh[3], Diana Zwahlen[3]

[1] Swiss Tropical and Public Health Institute, Basel, Switzerland

[2] University of Basel, Basel, Switzerland

[3] Department of Psychosomatic Medicine, University Hospital and University of Basel, Basel, Switzerland

[4] Division of Clinical Psychology and Cognitive Behavioural Therapy, International Psychoanalytic University, Berlin, Germany

[5] Division of Clinical Psychology and Epidemiology, Department of Psychology, University of Basel, Basel, Switzerland

[6] Institute of Pharmaceutical Medicine (ECPM), University of Basel, Basel, Switzerland

[7] Department of Health Canton Basel-Stadt, Division of Prevention, Basel, Switzerland
[8] University of Basel, Geriatric Psychiatry, University Department of Geriatric Medicine FELIX PLATTER, Basel, Switzerland
[9] University of Basel, Center of Old Age Psychiatry, Psychiatric University Hospital, Basel, Switzerland
[10] Centre for Primary Health Care, University of Basel, Switzerland
[11] Department of Psychiatry and Psychosomatics, Bethesda Hospital, Basel, Switzerland
[12] Department of Obstetrics and Gynecology, University Hospital and University of Basel, Switzerland
[13] St. Claraspital, Medical clinic, Basel, Switzerland
[14] University Department of Geriatric Medicine FELIX PLATTER, Basel, Switzerland
[15] Division of Internal Medicine, University Hospital and University of Basel, Basel, Switzerland;
[16] Department of Clinical Research, University Hospital and University of Basel, Basel, Switzerland
[17] Social Insurance Institution Basel-Landschaft, Binningen, Switzerland
[18] Foundation Rheinleben, Basel, Switzerland
[19] Department of Health Canton Basel-Stadt, Division of Addictions, Basel, Switzerland
[20] Department of Medicine, Division of Nursing, University Hospital Basel, Basel, Switzerland
[21] Direktion Pflege/MTT, University Hospital and University of Basel, Basel, Switzerland
[22] Department of Psychological Medicine, Institute of Psychiatry, Psychology and Neuroscience, King's College London, London, United Kingdom
[23] Sevogel-Apotheke, Basel, Switzerland
[24] Baselstädtischer Apotheker-Verband, Basel, Switzerland

[25] Department of Clinical Research, University of Basel, Basel, Switzerland

[26] University of Michigan School of Nursing, Ann Arbor, MI USA

[27] Swiss Heart Foundation, Bern, Switzerland

[28] University Hospital of Basel, Basel, Switzerland

[29] University Psychiatric Clinics (UPK), Department of Psychiatry and Psychotherapy, Basel, Switzerland

[30] IV-Stelle Basel-Stadt, Basel, Switzerland

[31] Swiss Academy for Psychosomatic and Psychosocial Medicine (SAPPM), Reiden, Switzerland

[32] Centre of Self-Help Basel, Basel, Switzerland

[33] Vereinigung der psychosomatisch tätigen Aerztinnen und Aerzte der Region Basel, Basel, Switzerland

[34] Psychotherapists Association of Basel VPB, Basel, Switzerland

[35] Department of Health Canton Basel-Stadt, Medical Services, Basel, Switzerland

[36] Liaisonpsychiatrischer Dienst, University Hospital Lausanne, Lausanne, Switzerland

[37] Institute of Psychology, RWTH Aachen University, Aachen, Germany

[38] Gyn. Social Medicine and Psychosomatics, University Hospital and University of Basel, Basel, Switzerland

[39] Department of Health Canton Basel-Stadt, Health Care, Basel, Switzerland

[40] Rheumaliga beider Basel, Basel, Switzerland

[41] Patientenstelle Basel, Basel, Switzerland
